# Experiences of an unconditional cash transfer intervention among young adults with first-episode psychosis in South Africa: qualitative inquiry of patients and their caregivers

**DOI:** 10.1080/17482631.2025.2576670

**Published:** 2025-11-16

**Authors:** Joyce Mlay, Vuyokazi Ntlantsana, Neliswa Gcabashe, Lise Jamieson, Thirusha Naidu, Busisiwe Siphumelele Bhengu, Lindokuhle Thela, Saeeda Paruk, Bonginkosi Chiliza, Jonathan K. Burns, Richard Lessells, Andrew Tomita

**Affiliations:** a Discipline of Public Health Medicine, School of Nursing and Public Health, University of KwaZulu-Natal, Durban, South Africa; b Health Economics and HIV and AIDS Research Division (HEARD), University of KwaZulu-Natal, Durban, South Africa; c Discipline of Psychiatry, Nelson R Mandela School of Medicine, University of KwaZulu-Natal, Durban, South Africa; d School of Nursing and Public Health, University of KwaZulu-Natal, Durban, South Africa; e Health Economics and Epidemiology Research Office, Department of Internal Medicine, School of Clinical Medicine, Faculty of Health Sciences, University of the Witwatersrand, Johannesburg, South Africa; f Faculty of Health and Life Sciences, University of Exeter, Exeter, UK; g Centre for the AIDS Programme of ResearchSouth Africa (CAPRISA), Durban, South Africa; h KwaZulu-Natal Research Innovation and Sequencing Platform (KRISP), College of Health Sciences, University of KwaZulu-Natal, Durban, South Africa; i Centre for Rural Health, School of Nursing and Public Health, University of KwaZulu-Natal, Durban, South Africa

**Keywords:** UCT, FEP, young adults, South Africa, qualitative, caregivers

## Abstract

**Introduction:**

Strengthening social protection through cash transfers has proven effective in reducing common mental disorders such as anxiety and depression. However, the acceptability of unconditional cash transfer (UCT) interventions, also known as Basic Income Support (BIS) in certain regions, for socially vulnerable young adults who have experienced first-episode psychosis (FEP) in sub-Saharan Africa, including South Africa, remains unknown. This qualitative inquiry explored the experience and acceptability of an unconditional cash transfer (UCT) intervention among patients with FEP and their caregivers.

**Methods:**

The study was conducted at government hospitals in KwaZulu-Natal Province, South Africa. In this descriptive phenomenological qualitative study, we aimed to interview 15 recipients of a UCT intervention with their caregivers based on convenience sampling. An interview guide was designed to explore recipients’ experiences with money utilization and budget decisions, as well as their views on preferred recipients, the effects of UCT, and their recommendations on how much money is needed to cater to their needs. Information saturation was achieved after interviewing ten FEP recipients and their caregivers. NVIVO 14 was used to analyze the data using interpretive phenomenology.

**Results:**

The UCT intervention was well accepted, with funds used for transportation to the hospital and purchasing groceries and food. Indirectly, UCT enhances family relationships and medication adherence and reduces patient and caregiver stress.

**Conclusion:**

The UCT intervention was acceptable and positively experienced by patients with FEP. This study highlights the need to enhance social protection mechanisms to support engagement in mental health treatment for FEP.

## Introduction

Psychosis is a relatively uncommon but highly debilitating psychiatric illness that manifests as a first episode of psychosis (FEP) in youth. This is an important life–course stage in which individuals establish peer networks, transition from family to independence, and explore their role in the world (Mackrell & Lavender, 2004). The onset of FEP during this period can significantly disrupt the development of social networks (MacDonald & Leary, 2005) and affect an individual’s life and goal attainment (Burns et al., 2011). Compared to other individuals, young people with FEP are often unskilled, unemployed, and socially isolated (Burns & Esterhuizen, 2008), resulting in a broad range of challenges that disrupt several functional domains (Watson et al., 2018).

The early phase of psychotic disorders offers a critical window during which timely intervention can positively influence the course of the illness (Kohler et al., 2023). Early intervention is a key management strategy for achieving clinical remission and better outcomes in patients with FEP (Jääskeläinen et al., 2013; Jordan et al., 2018). Evidence shows that early intervention is associated with less hospitalization, more rapid recovery, lower treatment resistance, and lower risk of relapse (Jordan et al., 2018). Furthermore, early intervention and support (Lally et al., 2017) improve social and occupational functioning and prevent long–term mental health issues (Jääskeläinen et al., 2013).

The continuity of mental health care is often disrupted by limited access to services, particularly in under–resourced areas (Isaacs et al., 2024). This challenge is further exacerbated by poor socio–economic conditions, which increase the burden on families and limit their capacity to seek and sustain ongoing access to mental health care (Patel et al., 2010). In South Africa (SA), mental health services, and particularly services for people with FEP, remain inadequate and often inaccessible (Shisana et al., 2024). Also, this is compounded by high levels of poverty, with approximately half of the youth living under the poverty line of R604 [$40] per month because of limited work opportunities and poor socioeconomic circumstances (Burns et al., 2011; Wilkinson et al., 2017).

Cash grants are one of the most effective interventions for alleviating poverty in low–and middle–income sub–Saharan African (SSA) countries, including SA, and are effective in relation to child health, family functioning, and youth mental health (Angeles et al., 2019; Kelly, 2019; Mitra, 2010; Nnaeme et al., 2020; Nnaeme, 2021; Zembe-Mkabile et al., 2015).

There is some evidence of the effectiveness of cash transfer interventions for common mental disorders, such as anxiety and depression (Leight et al., 2024; Pega et al., 2017; Zimmerman et al., 2021), including a recent meta–analysis that suggests they are effective in relation to youth mental health in low–and middle–income countries (Yoshino et al., 2023).

Cash grants can take various forms, including conditional cash transfers (CCT) and unconditional cash transfers (UCT) (Baird et al., 2013; Prencipe et al., 2021). CCT is given with specific conditions attached (e.g., school attendance), requiring adherence for continued access, while UCT is a payments without conditions or required actions (e.g., the Universal Child grant, disability grants) (Handa et al., 2015; Handa et al., 2016; Leight et al., 2024), the latter proving a less stressful or burdensome approach (Yoshino et al., 2023). Both are perceived as necessary and beneficial, addressing immediate consumption needs and contributing to long–term outcomes such as improving mental health (Pega et al., 2017).

Poverty is a key determinant in the onset and progression of psychotic disorders and strengthening social protection systems primarily through conditional or unconditional cash transfers can help address these socioeconomic challenges. Compared to the traditional biomedical approach for treating psychosis, which primarily targets individual symptoms through pharmacological and clinical interventions, both CCT and UCT may improve socio–economic status, giving more stability and the capacity to invest in mental health. However, the motivation for UCTs, especially for vulnerable populations (Levasseur et al., 2018), stems from the desire to empower individuals by having faith and trust in their agency, allowing them to make their own choices based on the principle of person–centered mental health care. We argue that setting specific conditions associated with CCT may be too complex to program for the strained healthcare setting, given that a person with serious mental illness has multiple and complex needs.

Pega and colleagues’ framework outlines the direct and indirect pathways through which the UCT can influence healthcare utilization. The direct pathways through which UCTs influence healthcare utilization include the reduction of financial barriers to accessing care, such as transport and service fees. The indirect effects operate through income, employment, and psychosocial improvements, enhancing individuals’ ability and motivation to seek and utilize health services (Pega et al., 2017).

However, the acceptability of unconditional cash transfer (UCT) interventions for socially vulnerable young adults who have experienced first–episode psychosis (FEP) in sub–Saharan Africa, including South Africa, remains unknown. This study explored the experience and acceptability of the UCT intervention among youth FEP and their caregivers in KwaZulu–Natal (KZN) Province, South Africa.

## Methods

### Study setting and design

This qualitative study was undertaken at government hospitals in Msunduzi Municipality in KwaZulu–Natal (KZN) Province, South Africa. The hospitals are located in a municipality with a population of 679,000 people, 80% of whom are Black Africans; over half live in rural areas, and the unemployment rate is more than one in three people (Conroy et al., 2016). This qualitative study employed a descriptive phenomenological approach to assess recipients’ and caregivers’ experiences of UCT. The methodological consideration of descriptive phenomenology entails exploring, analyzing, and describing a phenomenon while maintaining its richness (Matua & Van Der Wal, 2015).

### Study population and sample

This qualitative study is nested within a pilot randomized controlled trial (RCT), which aimed to assess the feasibility and acceptability of the UCT intervention. As such, study participants from the qualitative study were drawn from the intervention arm in the pilot RCT trial. The study participants were young adults experiencing a FEP who were seeking medical care in the selected study setting, either as inpatients or outpatients, with the following inclusion criteria: (a) positive FEP status, diagnosed by a psychiatrist under the ICD−11 criteria for psychotic disorders. (b) in remission, (c) on anti–psychotic medication for treatment for less than six months after initial psychiatric hospital discharge, (d) age 18−29 years, (e) speaking isiZulu [language spoke to in the study area] or English, (f) unemployed, (g) and have been resident in the Msunduzi area for ≥6 months to ensure we engage only residents of Msunduzi and restrict those who only use the hospital service. FEP is defined in this current study as based on the use of anti–psychotic medication for less than six months after the initial psychiatric hospital visit (Breitborde et al., 2009). Remission was defined as the absence of positive and negative symptoms at enrollment time or with mild symptoms (2 or less positive or negative symptoms assessed by the research assistant). The exclusion criteria were: (h) lack of written consent and capacity to participate in our study. The inclusion criteria for caregivers were 18 years or older, being able to speak isiZulu or English, and living with the UCT recipients for more than six months. For the current qualitative study, we interviewed 10 recipients from the UCT interventions arm (from 30) with their 10 caregivers based on convenience sampling methods (i.e., sampling technique where participants are selected based on availability). Although the original plan was to interview 15 recipients of UCT interventions with their 15 caregivers, we interviewed 10 recipients from the UCT interventions arm with their 10 caregivers, having successfully achieved saturation (i.e., no new information, themes, or insights are observed in the data).

### Description of study intervention

The participants in the UCT intervention arm received cash without any attached conditions. The study participant received a total of R4050 (US$270), which was paid out in 3 installments (i.e., once a month for three months). The first payment (R1,350) was distributed after the first interview, with two subsequent installments of the same amount at the end of months one and three, either in the participant’s household, at the hospital site, or at a convenient location. The amount of R1350 (US$90) per month (adjusted for 2022 inflation, rounded up) of the intervention was based on the South African Institute for Economic Justice (Saifaddin, 2021) and is valued at the upper–bound poverty line. The amount given is within the range of other program transfers, although the duration of transfers varies across studies (Angeles et al., 2019; Greene et al., 2017; Prencipe et al., 2021). Participants also received R150 cash for time reimbursement per study visit in accordance with the South African National Ethics Guidelines (Horn, 2008) as well as the standard of care (SoC), which included the refilling of anti–psychotic medication prescriptions, planning for the next visit, provision of counseling services where appropriate, and constant appointment reminders using short message service (SMS) or calls twice in the week before visits. The PI and research assistant administered cash payments to the participants.

### Data collection

Study participants were interviewed to explore experiences and acceptability of UCT through in–depth interviews. Study participants were interviewed twice to examine whether the phenomenon changed over time. A trained interviewer, fluent in English and isiZulu, informed the ten patients and ten caregivers about the data collection procedure and conducted face–to–face, audio–recorded interviews using a semi–structured interview guide. The core questions explored their experience of the UCT and its effects, its role in facilitating care and reducing poverty, how it influenced budgeting decisions and money utilization, the preferred recipient, and participants’ recommendations on how much money was required to meet their needs. We collected all demographic information for participants with FEP during the baseline interview of the RCT. Caregivers’ demographic information was collected during the qualitative interview. The first interview was approximately 40 min, while the second was approximately 30 min.

### Data analysis

Demographic information was analyzed descriptively for both groups of participants, and an interpretative phenomenological analysis (IPA) was employed to explore how they made sense of the intervention experiences (Smith & Nizza, 2022; Smith, 2024). Each audio–recorded interview was transcribed verbatim and, where necessary, translated into English and analyzed before the following interview (Ridders, 2018). NVIVO 14 software was used to manage the data, with three authors independently coding the text using an inductive approach (generating patterns, themes, or theories from the data itself). Subsequent transcripts were coded deductively (codes applied to the established theme), and the process was data–driven (Dhakal, 2022; Ridders, 2018; Vaismoradi et al., 2016). Using the interpretivist approach, clustered codes that expressed the same or similar ideas generated sub–themes and themes.

### Ethics

The study was approved by the Biomedical Research Ethics Committee (BREC, 00004117/2022) and the KZN Department of Health (KZ_2002209_033) and registered with the South African National Clinical Trial Registry (#DOH- 27- 092022- 5894). All participants provided written informed consent, and to ensure confidentiality throughout the study, participants were identified by a unique number and not by their names.

### The rigor of qualitative inquiry

A qualitative research approach ensures trustworthiness by adhering to dependability, credibility, transferability, and confirmability criteria (Schwandt et al., 2007). Credibility was ensured by triangulating the data from the patients with that of their caregivers to obtain corroborating evidence by enhancing the quality of information from different sources. We also ensured credibility by collecting data longitudinally to determine whether the phenomenon had changed over time. Dependability was ensured using a specific interview guide for the patients and caregivers, conducting inter–coder analyzes, and debriefing the initial results to the study team. Confirmability was ensured by keeping interview notes, audio recordings of the interviews, and verbatim transcriptions that enabled quotes to be presented. Finally, a thick description of the results was reviewed with respect to other studies in the same context to ensure transferability.

## Study findings

### Socio–demographic and other characteristics

Most of the UCT recipients were male (8 out of 10), 50% were aged 21–25 years (5 out of 10), and 6 had completed secondary school education. All were single, most were black (9 out of 10), nearly half lived in rural areas (4 out of 10), most did not receive grants (7 out of 10), and seven were diagnosed with schizophrenia (Table I). Most caregivers were female (8 out of 10), 4 were in the age range between 40 and 50 years, and had completed secondary school education. Only 3 were married, the majority were unemployed (8 out of 10), and the parents of the patients (7 out of 10) (Table II).

**Table I.** Socio–demographic and other characteristics of the study participants (Patients).

Characteristics	*n* = 10
Gender	
Male	8
Female	2
Education	
Primary school completed	4
Secondary school completed	6
Age	
18−20	3
21−25	5
26−29	2
Race	
Black	9
Indian	1
Marital status	
Single	10
Residence*	
Urban	2
Township	3
Rural	4
Informal settlement	1
Diagnosis	
Schizophrenia	7
Substance–induced psychosis	2
Bipolar	1
Individual grants	
Child support grant	1
Unemployment grant R350	2
No grant	7
Household average income**	
Below food poverty line	5
Food poverty line	1
Upper–bound poverty line	4

* Participant residential data were self–reported.

** Categories for a household average income; food poverty line (FPL) R614, Lower bound poverty line (LBPL)R819, and Upper bound poverty line(UBPL)R1335 for individual level (Abrahams & Lund, 2022).

**Table II.** Caregiver characteristics (*n* = 10).

Gender	
Male	2
Female	8
Race	
Black	9
Indian	1
Marital status	
Married	3
Single/Divorced	7
Education	
Primary school completed	5
Secondary school completed	5
Employment status	
Employed	2
Unemployed	8
Age	
<40	3
40−50	4
51−60	3
Relationship with the patient	
Father/Mother	7
Aunt/Uncle	1
Sister/brother	2

The themes that emerged from the analysis were based on all participants’ views, other than the first theme, which was based on caregivers’ opinions only, namely, the acceptability of UCT. Other themes included the effects of UCT, decisions on the use, the use of the money, suggestions on who should receive the money, and the adequacy of the amount.

**Theme 1: Caregivers’ opinions on the acceptability of UCT**

Regarding caregivers’ perspectives on the acceptability of UCT, they generally expressed a positive attitude. Even though the funds were given with no conditions, they found that the patients wisely used the money, highlighting several key aspects. They felt that patients accepted the responsibility of looking after the money and making decisions about how it was spent for their benefit and family, such as buying food. The financial assistance also offers support for transportation for themselves and those accompanying them to the hospital. They also allocated a small amount of money to purchase food because taking medication on an empty stomach can be challenging.

*“It would help them to have money for transport, especially if they have multiple clinic appointment dates in a week or a month. Furthermore, since they have this condition and have recently been diagnosed, some of them may need someone to accompany them to their clinic and hospital visits. This means they need transport money for two people. Also, they wake up very early in the morning when going to the clinic to avoid being stuck in a long queue. The money also helps them buy food [while waiting for services] and not take their medication on an empty stomach.”* **(Caregiver 10, female 45 years)**

Other caregivers felt that receiving help through the UCT offered more than the transport money for patients. They emphasized that it could address various other needs, such as money to buy cosmetics and clothes, which helps patients maintain a sense of self–esteem and appearance, enabling them to feel better about themselves and enhance their overall well–being.

*“Also, they can buy cosmetics to be clean and look good. You know, most people tend to assume that people with this type of illness are mad or crazy, but that’s not the case**.**”* **(Caregiver 7, female 58 years)**

Some caregivers shared that receiving help through the UCT encouraged patients to remain engaged in their care. They explained that there were instances when the patients needed assistance, but they could not help them because they did not have the means to buy what they wanted. As a result, some think their parents do not love them, but with the UCT, patients can independently address their needs by receiving direct assistance.

*“I think it would encourage them, as sometimes they may ask for something from you as a parent, while you don’t have the means to do it. Then they assume you don’t love them, but that’s not the case; you can’t do it for them now due to financial constraints. So, receiving money could greatly help them; they can buy even a loaf of bread to take their medication, having eaten something.”* **(Caregiver 8, female 56 years)**

The caregiver explained that receiving assistance through UCT alleviates their stress, as one of their concerns is often working out how to afford the transport cost for themselves and the patients. They frequently worry about sourcing money for transport, which adds to their stress; UCT payment provides them with a sense of relief, as it eliminates the need to find money for transportation expenses.

*“It helps a lot; I have seen the impact of this money on their lives. It has made a huge difference, as I sometimes worry when he has to go to the clinic, knowing I don’t have money. Then I would start stressing, trying to figure out where I am going to get the money or who I can borrow it from because he has to collect his medication.”* **(Caregiver 7, female 58 years)**

**Theme 2: Effects of UCT**

Four sub–themes related to the effect of UCT emerged, as shared by patients and caregivers. The sub–themes were empowering the continuity of patient care, improving medication adherence, rebuilding identity and acceptance, and improving family relationships.

***Sub–theme 1. Empowering continuity of patient care***

Both patients and caregivers shared that the patients may have multiple clinic appointments scheduled close together, necessitating additional funds to be available for unexpected trips. As a result, the patients were able to attend all the planned appointments without worrying about finding money or taking it away from other household needs, such as buying food.

*“The money was helpful, but the challenge we face is that the clinic schedules many appointments on different dates that are not too far apart. They are taking blood samples on the 20th of the month. The next date may be the 28*^*th,*^ *when he needs to go back and collect medication. Now that is a lot of money for transport.”* **(Caregiver 1, female 37 years)**

*“The money helped me a lot because he was also inspired to go to the clinic to collect his medication, and I was not worried knowing that he had money for transport.”* **(Caregiver 3, female 42 years)**

*“I used to struggle with not having transport money, but having received this money from you, I wasn’t worried about not having transport fare for when I needed to go to the clinic*.” **(Patient 8, male 29 years)**

Some patients mentioned that their family members were sometimes occupied with other responsibilities and had to rely on external help and pay someone to accompany them to their appointments. The financial support provided meant they did not have to worry about finding the money, making it easy to pay for the one to accompany them.

*“I could pay the person who accompanied me to the clinic. Some days, my aunt and grandmother would be busy or need to go somewhere else, so they could not go with me to my appointment. Therefore, I would need to pay someone to help me, just as I did the last time, because I could not afford to miss my appointment. I also used the money for my other needs.”* **(Patient 1, male 25 years)**

***Sub–theme 2. Improving medication adherence***

Caregivers and patients shared that financial assistance helped them adhere to taking their medication, as it ensured that they had sufficient food, preventing them from sometimes skipping their medication dose to avoid taking them on an empty stomach. This support helped them to follow their medication regimes more consistently and knowing that they would receive financial help encouraged them to continue their treatment. This reassurance reduces stress and uncertainty associated with managing health and finances, improving medication adherence.

*“Yes, it helped a lot because I sometimes wouldn’t take my medication at all to avoid taking it on an empty stomach.”* **(Patient 9, male 23 years)**

Although the intervention involved unconditional cash transfers with no specific requirements for participants to continue receiving the funds, one caregiver noted that the patient was naturally motivated to adhere to the treatment simply because he knew he would receive financial support.

*“I can say that knowing that he was going to get money again encouraged him to take his medication better than he normally does.”* **(Caregiver 2, female 37 years)**

***Sub–theme 3. Rebuilding identity and acceptance***

Some patients disclosed how financial assistance provided them with the means to enhance their self–care. They emphasized that they could now afford to buy personal hygiene products and new clothes and shoes, making them appear clean. They also noticed that this resulted in a positive change in how people perceived them, restoring their sense of belonging within their communities.

*“It made a huge difference. It helped to restore my dignity as a person because people were isolating me, viewing me as a crazy or mad person, but after seeing me clean and wearing new clothes, they started accepting me as one of them again. Even my friends who were looking at me funny came back, and we are friends again.”* **(Patient 3, male 23 years)**

*“Being unemployed means that you end up being undermined by people. Receiving this money meant I was able to buy myself clothes, and that made me feel better about myself.”* **(Patient 9, male 23 years)**

***Sub–theme 4. Improving family relationships***

A few participants believed that UCT improved their family relationships as they no longer relied on their relatives for money. Some noticed improvements in their family relationships as they could better support their needs and did not have to ask for help, even though their relationships were good before the intervention.

*“Our relationship was OK without the money, but I can say it got even better since I could also help out here at home with the help of this money.”* (**Patient 8, male 29 years)**

Additionally, all patients and caregivers shared that they did not experience any adverse effects, such as theft incidents or fights about money.

*“There has been a big difference; sometimes, he would demand money and fight me to give it to him. But that hasn’t happened since getting money from you; it’s been very peaceful. Even after he had finished his money, he asked in a very polite manner when he needed it. He is better, and he hasn’t fought with anyone.”* (**Caregiver 8, female 58 years)**

*“No, there were no arguments, and I respect my family. They were happy that I received this money and that I bought groceries and clothes.”* **(Patient 3, male 23 years)**

**Theme 3: Decisions on the use of the money**

Regarding who made decisions regarding the allocation of funds obtained from the UCT intervention, most indicated that they jointly (caregiver and patient) agreed on how to use the money. Furthermore, the caregivers said they did not allow the patients to be fully responsible for deciding how to use the money to avoid misuse. Some patients indicated a weakness related to poor financial management, and to avoid wasting money, they preferred that the caregivers keep it and give it to them when needed.

*“We would all discuss until we reached an agreement about the things needed here at home. Sometimes, they would ask me what to do, but honestly, they are the ones who always know about such things, including food. They are the ones who cook for me, so I waited to hear from them if they needed anything, and I would then go and buy it. I didn’t dictate to them about how to use the money.”* **(Patient 1, male 25 years)**

*“I was afraid that I would overspend it or use it recklessly”* **(Patient 8, male 29 years)**

*“Uhm, I have had financial issues before; to be honest, I am not good at keeping money. Also, she doesn’t trust me since I drink alcohol”* **(Patient 6, female 28 years)**

*“Him and my mom, his grandmother, decided how the money should be used. He would suggest what needed to be bought and discuss with his grandmother before concluding.”* **(Caregiver 1, female 37 years)**

One caregiver shared that they allowed the patient to decide how to use the money to avoid conflict. As the patient was recently discharged from the hospital, they did not want to cause conflict, which could trigger a relapse.

*“We tried to decide, or rather to offer guidance, but we ended up not seeing eye to eye. He didn’t seem to like having someone else decide for him. Since he had recently gotten out of the hospital, I didn’t want to trigger him in any way, given his condition. I just let him be. Therefore, he ended up deciding on his own”* **(Caregiver 8, female 56 years)**

**Theme 4: The use of the money**

This theme summarizes the use of money obtained in the form of UCT interventions among patients and caregivers. Both caregivers and participants shared that the money received was used for various essential needs, including transport, ensuring that they could attend scheduled hospital visits, helping with groceries, especially food, and that some used the money to buy cosmetics.

*“Yes, the money was of great assistance to him and us as his family. When he finished his medication, he could go to the clinic and collect it with the help of this money. I am so grateful for this intervention, especially because I wasn’t aware of such studies that required this type of help. As parents, we are happy”* **(Caregiver 8, female 56 years)**

*“The money helped greatly as I could buy groceries for my family. The day you guys visited me for the first time, there was no food in the house, so we bought food. I also had a scheduled clinic visit, so the money helped me to pay for transport.”* **(Patient 1, male 25 years)**

One participant with a child narrated that the money enabled her to meet her child’s needs as she struggled after having to resign from work and not having an income.

*“I stayed for a long time at the hospital, so I had to resign from work; that way, I had no source of income. The day I got discharged, I met with you, and you gave me the money; the first thing I did was to buy my baby’s things, as that is what my mom struggled with a lot when I was in the hospital. The baby formula was finished, so we passed by the shops and bought it and other things. We also used that money to pay for the transport fare that day. I also gave some of the money to my mom so she could keep it for me to use whenever I must attend hospital appointments and check-ups; right now, I don’t have money as I am unemployed. I have an appointment on Monday, the 4th, and will take that money to cover my transport fare.”* **(Patient 6, female 28 years)**

**Theme 5: Suggestions about who should receive the money**

Regarding suggestions about who should receive the money for future interventions, most caregivers suggested that the family or caregiver should receive the money, as it is often difficult to trust the patient’s behavior regarding financial management. The caregivers expressed concerns that patients might misuse the funds because of issues such as substance use.

*“He may misuse the money on a lot of things that are not so important. A parent can receive money, show it to him, and then ask him what he needs or wants to do with the money. That way, they would have ensured he didn’t spend it recklessly.”* **(Caregiver 7, female 58 years)**

*“Could receive the money on her behalf and then discuss with her so that we reach a mutual agreement on how to use that money.”* **(Caregiver 6, female 46 years)**

*“Maybe he might make decisions unsuitable for his state of mind, as he is still a child. He might not think of how the money must be spent and how it should assist him.”* (**Caregiver 9, male 35 years)**

*“I don’t trust that he has completely quit drugs.”* (**Caregiver 3, female 42 years)**

Two caregivers suggested that the patient receive money to avoid conflict and give them a sense of autonomy over how it was spent, with the family or caretakers only guiding them on its use. Personal responsibility would provide patients with a better understanding of how much they had, what it had been spent on to be finished, and when it was finished, being unable to blame anyone else if it was not spent wisely.

*“It better be a family member (caregiver); however, their aggressive behavior may sometimes upset them. They may feel like they are being controlled or that their family members are ripping them off. I think he may receive it himself, but as an elder, I would need to guide him, so he doesn’t misuse the money. Him receiving the money and involving him in making decisions regarding how it should be used will help him be aware when it is finished, so he does not fight us wanting his money.”* **(Caregiver 1, female 37 years)**

*“I don’t want him to think or feel like we are treating him as a mad person or belittling him in any way, just because he has this mental condition. However, I should be there to guide him as his parent.”* **(Caregiver 8, female 56 years)**

**Theme 6: Adequacy of the amount**

Most of the participants were satisfied with the amount of money given, saying that, as they did not work for it, they thought it was adequate to meet their needs and were grateful. They saw the benefits and felt that it enabled them to meet their transport needs and ensure that they ate adequately, which helped them take their medication regularly. Some were aware of the possibility of obtaining a disability grant; the money from the current study was prepared for how the fund would be spent productively to keep them healthy.

*“I still say R1350 is enough, as I don’t have many needs. One of the doctors in the hospital mentioned that I need to apply for a government disability grant, as he doesn’t think I will ever be employed anywhere due to the severity of my sickness. He advised me to wait a little longer as they continued to run more tests.”* **(Patient 1, male 25 years)**

*“The way I see it, it is enough. I could use R1000 for groceries and the R350 for transport fare.”* **(Patient 6, female 28 years)**

However, one participant shared that the amount given was insufficient to cater to his needs and suggested that it should be R1800-R2000, although he did not indicate what it would be spent on.

*“The money is insufficient for all my needs; perhaps it could be increased a little to between R1800 to R2000.”* **(Patient 8, male 29 years)**

Although we ensured credibility by collecting data longitudinally to determine whether the phenomenon had changed over time, we did not observe any changes in the above–mentioned response.

## Discussion

This qualitative study explored the experience and acceptance of UCT receipt among young adults living with FEP and their caregivers. The three major findings from the support provided through the UCT intervention are as follows. First, the caregivers accepted the intervention and found it beneficial in helping patients stay in care. Second, funds were effectively used to address immediate needs, such as transportation to the hospital, buying groceries, and child support, with additional indirect effects on medication adherence and family relationships. Finally, most participants expressed satisfaction with the amount of money provided, agreeing that it was sufficient to cover the essential expenses.

Theme 1 examined the acceptability of the UCT intervention exclusively from the caregivers’ perspectives and revealed that they perceived the intervention as beneficial for the young patients. Caregivers emphasized how the financial support contributed positively to the patients’ overall well–being. Previous studies have demonstrated that caregiver satisfaction with cash transfer programs is a critical factor in the uptake and success of health interventions (Swartz et al., 2022). Moreover, systematic reviews and meta–analyzes evaluating the impact of poverty alleviation programs on mental health underscore the significance of intervention acceptability, whether assessed prospectively or retrospectively (Zaneva et al., 2022). Although qualitative studies on UCTs in mental health are limited, similar findings from HIV–related research have shown that caregivers often express high levels of satisfaction with UCTs, particularly as these interventions help to ease the financial and emotional burden of caregiving (Swartz et al., 2022). “The intervention highlights a potential need for government–led strategies to provide short–term support to bridge the gap to long–term social support, such as a disability grant for those unable to recover from the illness, or to employment, education, or training”.

Regarding theme 2, the effects of the UCT, the patients and caregivers shared that the financial support provided by the intervention helped them continue with mental health care, specifically to attend scheduled visits, as they were motivated by the availability of funds to cover transport costs and food. Evidence from previous studies indicates that continuity of care during the early stages of psychosis is critical for improving clinical outcomes, including enhanced social functioning and a reduced risk of relapse (Fusar-Poli et al., 2017). Similarly, other cash transfer studies which does not focus on psychosis, such as those targeting pregnant women, have demonstrated improved attendance at antenatal visits and enhanced continuity of care in HIV studies (Humphries et al., 2017; Handa et al., 2015; Naidoo et al., 2017; Pettifor et al., 2016. Although South Africa allocates only about 5% of its overall health budget to mental healthcare, this proportion remains higher than that of many neighboring countries (Docrat et al., 2019). Participants in this study reported that they did not have to pay direct costs for essential mental health services, including hospital admission, psychoeducation and antipsychotic medication. This suggests that the main financial barrier lies not in direct healthcare costs but in covering indirect costs, particularly transportation. In South Africa, the key mental health services are offered for free in government hospitals, unlike many other African countries, such as Ethiopia, Tanzania, and Kenya, where cash transfers may be utilized differently, due to the requirement to pay for mental health services (Shayo et al., 2016; Teshager et al., 2023; Toroitich et al., 2022).

This financial assistance not only facilitated access to care but also significantly improved medication adherence, as it enabled them to have the resources to purchase food, which is essential for effective medication intake. Research has consistently shown that food insecurity compromises adherence and increases the risk of relapse in individuals with psychosis (Mlay et al., 2024; Sariah et al., 2014). Comparable evidence from HIV–related interventions demonstrates that cash assistance can significantly improve the uptake of antiretroviral therapy (ARVs) by reducing economic barriers to care (Shelus et al., 2018).

In addition, caregivers and patients noted that having adequate funds reduced stress and burden on families, fostering better family relationships and understanding. Research indicates that strong family support plays a crucial role in promoting an understanding of a patient’s condition and the importance of treatment. This, in turn, enhances medication adherence and ensures continuity in mental healthcare and reduces relapse (Jiang et al., 2024). Similar findings on the positive impacts of cash transfer on family relations have been reported, in a study evaluating the impact of cash transfers from the effects of cash transfer grants in Zambia. (Handa et al., 2014).

Theme 2 also identified that patients and caregivers emphasized how the cash incentives from the intervention contributed to rebuilding their identity and acceptance. Financial support enables patients to afford clothing, shoes, and cosmetics, significantly improving their appearance and self–presentation. This transformation was crucial in altering how others perceived them, having previously been marginalized and labeled as “mad.” These individuals have experienced a shift in societal attitudes, allowing them to connect better with their peers and community members. Previous research shows that improved appearance enhances self–esteem and confidence, essential for social interactions and integration (Lannin et al., 2015). When patients see themselves as being treated more positively by others, they can reduce their internalized stigma and improve their overall mental well–being (Simbayi et al., 2007). Similar evidence from a systematic review of qualitative studies highlights the positive role of financial support provided through cash transfer interventions in country programs. These interventions were found to help mitigate stigma by enabling beneficiaries to manage their appearance and self–care better, enhancing their sense of dignity and social acceptance. (Yoshino et al., 2023). By alleviating visible signs of poverty and promoting improved self–presentation, cash transfers reduced the social stigma often associated with illness and economic hardship.

The responses to theme 3 (decisions on the use of money) indicated that caregivers often guided decision–making regarding the use of provided funds, particularly as some patients struggled with financial management. This aligns with other studies that advocate family involvement in managing financial assistance for young people (Ingoldsby, 2010). However, caregivers may sometimes have personal interests that could lead to conflicts of interest, and assessing a patient’s ability to manage finances can be challenging. In addition, some caregivers suggested that allowing patients to make financial decisions could prevent conflict and promote autonomy. Also, evidence shows that providing financial guidance to individuals with mental health conditions is essential in preventing the misuse of funds, particularly in purchasing substances (Herman et al., 2011).

Theme 4 focused on the use of money young adults and their caregivers reported that the funds primarily were for transport to attend scheduled hospital visits, which supported continuity of mental health care. The money was also commonly used to purchase groceries, particularly food, to improve medication adherence. One participant shared that the cash transfer allowed her to buy baby formula for her child, easing the stress associated with childcare. These findings align with evidence from evaluations of cash transfer programs aimed at improving educational outcomes, which show that such financial support is often used to meet essential needs such as food and school–related expenses and contributes to better school attendance and reduced dropout rates.

Regarding themes 5 (suggestions about who should receive the money) and 6 (adequacy of the amount), most caregivers and patients suggested that the patients should receive the money, but the parents should guide them on how to use the money; moreover, most patients were satisfied with the current amount of money given with one proposing an increase of the monthly amount to R1800-R2000, comparable to the disability grant (Kelly, 2013). The South African government introduced the COVID−19 Social Relief of Distress (SRD) grant of ZAR 350 in April 2020 to address the socioeconomic challenges caused by the pandemic. The SRD grant provided crucial immediate support to vulnerable individuals, helping them cover essential expenses, such as purchasing food and other basic needs (Venter et al., 2024). This is similar to the immediate support provided in the form of UCT, evidenced by one participant with a child who resigned from work due to her illness; the cash received from this intervention enabled her to meet her child’s needs. Notably, not all patients in this study benefited from disability grants, and only one participant was advised by their doctor to apply. This highlights the necessity for hospital social workers to actively assist patients in navigating the disability grant application process early to ensure timely receipt of these funds, which could significantly aid in meeting their needs and improving their outcomes.

Although this study found the intervention acceptable, in SA, the concept of deservingness with social grants is often linked to specific categories of vulnerability, including individuals with disabilities, the unemployed, older persons, and caregivers of children under the age of 18. These groups are legitimate recipients due to their limited capacity to engage in full economic participation and their need for assistance to meet basic living requirements. In this study, we engaged unemployed young adults experiencing FEP. However, eligibility for disability grants in SA requires medical validation of incapacity by a certified healthcare professional (Kelly, 2013).

### Limitation

There are several limitations to note. This study included only patients and their caregivers; the scope of future research should be broader and include health workers. We did not include participants from the control group, which limited our ability to compare experiences between groups. The study was conducted only in certain areas of KZN Province, and the results may not be generalizable to other South African provinces or SSA. Lastly, this study explored only the short–term experiences of the cash transfer. There is evidence that the impact of cash transfers persists beyond the program duration, and a future larger trial should incorporate evaluation of those longer–term impacts.

### Strength

Notwithstanding the above limitations, this study demonstrates that the UCT Intervention is perceived as positive and acceptable for young adults with the FEP.

## Conclusion

This qualitative study demonstrates the need to enhance social protection mechanisms to support mental health treatment engagement for FEP**.** The UCT intervention suggests that providing people with practical means to manage their health status, including patients with FEP, may improve continuity of care and family relationships, rebuilding identity and acceptance, and improve medication adherence. It also provides encouraging evidence that UCT intervention may be an important strategy for improving mental health outcomes for young people with FEP in this resource–constrained context, shifting the focus from purely pharmacological approaches to more holistic care strategies. The insights derived from this study can be used to develop a definitive randomized control study, which can provide more evidence of the effectiveness of UCT as an intervention.

## Acknowledgements

The authors acknowledge all the participants in this study. The research assistants from the PSYMAP–ZN for help in the recruitment process and excellent research assistance.

## Supplementary Material

Supplementary Material Famlly_interview_guide_UCT

## Data Availability

Data can not be shared in the public domain because we did not get permission from participants to share it publicly. Data are available from the University of KwaZulu Natal Institutional data access/UKZN Biomedical Research Ethics Committee. Contact BREC@ukzn.ac.za for researchers who meet the criteria for access to confidential data. Data will be kept for at least three years from the completion of the study.
