# Supplementary material for: Experiences of an unconditional cash transfer intervention among young adults with first-episode psychosis in South Africa: qualitative inquiry of patients and their caregivers
Source: Int J Qual Stud Health Well-being. 2025 Nov 16;20(1):2576670. doi: 10.1080/17482631.2025.2576670 (PMC12624895; doi:10.1080/17482631.2025.2576670)
Supplement: Supplementary Material — Famlly_interview_guide_UCT [file ZQHW_A_2576670_SM0905.docx]

FAMILY MEMBER INTERVIEW GUIDE.

EXPERIENCE OF UNCONDITIONAL CASH TRANSFER AMONG FAMILY MEMBER WITH YOUNG ADULT WITH FIRST-EPISODE PSYCHOSIS.

STRUCTURE -INTERVIEW GUIDE Participant ID.

Introduction

**Perception.**

Do you think Unconditional cash transfer, will help young adults with first episode psychosis?

Probe: remain in care, poverty alleviation?

**Experience**

1. Did the cash transfer received help you to take care of the young adults with first episode psychosis in your family?

Probe: remain in care? Poverty alleviation?

1. Who decides on how to use the money

Probe; Family, friends, relatives, or patient.

1. How did the recipients use the money?

Probe; for food, transport, hospital needs, alcohol and drugs

1. From your experience, who should receive the money?

Probe, patient, family or relative and why?

1. What difference does this transfer made to your family?

Probe: How does this grant impact your life?

1. What changes in your life since you receive the grant?

Probe, did the grant help you to continue/ remain with the care?

**Effects of unconditional cash transfer intervention**

1. Did the Unconditional cash transfer affect your relationship?

- Probe; social relationship with family, friends, and community?

1. Has there been an adverse outcome after receiving the transfer?

- Probe; Any threat or stilling of the cash transfer or conflict within a family
- Do you know of any safety incidences reported after cash distribution? Explain.

**Is there any recommendation for the transfer?**

1. What is the view about the cash value of R1350 per month?

- Probe is the money given enough to meet your needs. Groceries and attending the scheduled follow-up clinic?

1. What is the appropriate time for transfer to help the patients?

Probe: immediate after discharge? During first follow-up visit after discharge and why?
